# *Lactococcus lactis* mutants resistant to lactococcin A and garvicin Q reveal missense mutations in the sugar transport domain of the mannose phosphotransferase system

**DOI:** 10.1128/spectrum.03130-23

**Published:** 2023-12-04

**Authors:** Marco J. van Belkum, Tamara Aleksandrzak-Piekarczyk, Tess Lamer, John C. Vederas

**Affiliations:** 1 Department of Chemistry, University of Alberta, Edmonton, Alberta, Canada; 2 Institute of Biochemistry and Biophysics, Polish Academy of Sciences (IBB PAS), Warsaw, Poland; University of Saskatchewan, Saskatoon, Saskatchewan, Canada

**Keywords:** bacteriocins, resistance, man-PTS, lactococcin A, garvicin Q, fusion protein, mutational studies, sugar

## Abstract

Lactococcin A is a bacteriocin from *Lactococcus lactis* that permeabilizes the membrane of sensitive lactococcal cells and requires the presence of the membrane-bound components IIC and IID of the mannose phosphotransferase system (man-PTS). Recently, it was reported through cryo-electron microscopy analyses of man-PTS and several bacteriocins fused to a maltose-binding protein, including lactococcin A, that these bacteriocins create pores by inserting themselves between the Core and Vmotif domains of man-PTS. In our study, we obtained a dozen spontaneous mutants of *L. lactis* IL1403 resistant to lactococcin A. All but one of the mutants of IL1403 have mutations located in the genes encoding the IIC or IID proteins. These mutations also resulted in resistance to garvicin Q, a bacteriocin from *Lactococcus garvieae* with a broad inhibition spectrum and very little sequence homology to lactococcin A. Missense mutations were found in the sugar transport domain of man-PTS of bacteriocin-resistant IL1403 mutants, which also impeded the uptake of mannose. When lactococcin A, garvicin Q, or pediocin PA-1, an anti-listerial bacteriocin, were fused to a maltose-binding protein, we observed reduced or no antibacterial activity. Taken together, the precise mechanism of action of bacteriocins using the man-PTS remains to be fully understood.

**IMPORTANCE:**

Many bacteriocins target the sugar transporter mannose phosphotransferase system (man-PTS) to exert their antibacterial activity. The elucidation in recent years of the structure of man-PTS has facilitated our understanding of how bacteriocins might interact with the receptor and which domains of the transporter are involved in bacteriocin resistance. Here, we show that missense mutations in the sugar-binding domain of the man-PTS not only impede the uptake of sugars but also prevent the antibacterial activity of the bacteriocins lactococcin A and garvicin Q.

## INTRODUCTION

Many, if not most, bacteria produce ribosomally synthesized antibacterial peptides. These peptides, termed bacteriocins, enable the bacteriocin-producing bacteria to compete in an ecological niche by limiting the growth of other bacteria (1). Bacteriocin producers normally only kill closely related bacteria, but some bacteriocins have a broad inhibition spectrum. Lactic acid bacteria (LAB) that produce bacteriocins have become of great interest in preventing food spoilage or inhibiting the growth of food pathogens, as LAB are generally regarded as safe for use in food products (2). Given the rise of antibiotic-resistant bacterial strains, bacteriocins are also considered as alternatives to antibiotics (3). Although the classification of bacteriocins remains a topic of discussion, bacteriocins from Gram-positive bacteria can generally be divided into three major classes: class I consists of post-translationally modified peptides (RiPPs), class II includes small post-translationally unmodified bacteriocins, whereas large, heat-labile bacteriocins are grouped into class III (4).

Lactococcin A is one of the first class II bacteriocins that had its complete genetic operon cloned, expressed, and sequenced, as well as its mode of action studied in detail (5 – 7). The bacteriocin is produced by *Lactococcus lactis* and has a narrow antibacterial activity inhibiting only *L. lactis* strains (8). Lactococcin A is able to permeabilize the membrane of sensitive cells resulting in the dissipation of the proton motive force, leakage of ions and amino acids, and ultimately cell death (6). Although a membrane receptor was postulated, it was not until more than a decade later that the mannose phosphotransferase system (man-PTS) was identified as the target of lactococcin A (9). The man-PTS is able to take up mannose using several cytoplasmic and membrane-bound proteins with concomitant phosphorylation of the sugar in a phosphorylation cascade using phosphoenolpyruvate as the first phosphoryl donor (10). Other sugars can also be transported by the man-PTS, and this system is the main transporter of glucose in many lactic acid bacteria (11). The man-PTS is unusual compared to other PTS transporters by having a permease that consists of two proteins, IIC and IID, instead of one. Expression and deletion experiments of subunits IIC and IID in Gram-positive bacteria showed that these proteins were involved in the recognition of various class IIa bacteriocins and might act as a bacteriocin receptor (12 – 14). Class IIa bacteriocins, also termed pediocin-like bacteriocins, contain a conserved N-terminal YGNG motif and at least one disulfide bridge (4). Although lactococcin A uses the same receptor as class IIa bacteriocins, it does not contain an YGNG motif, has no disulfide bridges, and is often referred to as a class IId bacteriocin. In recent years, several other class IId bacteriocins have been identified that use the man-PTS as a receptor, including lactococcins B and Z, BacSJ, garvicins A, B, C, and Q, ubericin K, and angicin (9, 15 – 20). However, it should be noted that not all class IId bacteriocins use the man-PTS as a receptor (21).

Recently, the structure of the man-PTS has been elucidated by cryo-electron microscopy (cryo-EM) (22). It was shown that the *Escherichia coli* man-PTS consists of a trimer with each protomer containing an IIC and an IID protein. The Vmotif domains of IIC and IID are responsible for interlocking the various subunits into the trimer configuration, whereas the Core domains of IIC and IID function as the sugar transport domains of the man-PTS (22). Subsequently, the man-PTS structures of *Listeria monocytogenes*, *Lactobacillus sakei,* and *L. lactis* were reported in complex with the class IIa bacteriocins pediocin PA-1 and sakacin A, and lactococcin A, respectively (23 – 25). These cryo-EM results were obtained with bacteriocins that were fused to a maltose-binding protein (MBP) at the N-terminus of the bacteriocin. The cryo-EM structures suggested that these bacteriocins form pores by inserting themselves between the Core and Vmotif domains of the man-PTS. In addition, when the man-PTS/bacteriocin complex was studied in combination with bacteriocin immunity proteins, the immunity proteins were found to be inserted into the bacteriocin-induced pores from the cytosolic site thereby blocking the leakage of compounds (24, 25).

In this study, we report the characterization of spontaneous *L. lactis* mutants that are resistant to lactococcin A. Genetic analyses of the genes encoding the man-PTS from a dozen lactococcin A-resistant mutants revealed that 11 mutants contain mutations in the *ptnC* or *ptnD* genes encoding IIC and IID, respectively. The *L. lactis* mutants are also equally resistant to garvicin Q. Surprisingly, missense mutations were observed in the sugar-binding domain of the man-PTS that are located at some distance from the cleft between the Core and Vmotif domains, where bacteriocins are postulated to insert themselves. These mutations also impeded the uptake of sugars, such as mannose and glucose. The antimicrobial activity of bacteriocins fused to a maltose-binding protein was tested, and substantially reduced inhibition of the growth of sensitive bacteria was observed. This suggests that the bacteriocin-maltose-binding protein fusions used in previous cryo-EM structural work may not fully represent native bacteriocin-receptor interactions.

## RESULTS AND DISCUSSION

### Isolation and genetic analyses of bacteriocin-resistant mutants

During the course of our previous work on an improved expression and isolation procedure of small peptides, we observed small colonies of bacteriocin-resistant *L. lactis* IL1403 when spot-on-lawn assays were performed using lactococcin A (26) (data not shown). The appearance of spontaneous lactococcin A-resistant colonies of *L. lactis* IL1403 has been noticed before (27, 28). Kjos et al. obtained four different lactococcin A-resistant IL1403 mutants but did not detect mutations in the *ptnABCD* genes that encode the man-PTS of *L. lactis* (28). Instead, it was discovered that the gene expression level of *ptnABCD* was downregulated in three of the four mutants resulting in a 16- to 67-fold increase in resistance to lactococcin A. In our study, we isolated 12 colonies of spontaneous lactococcin A-resistant *L. lactis* IL1403 cells. The *ptnC* and *ptnD* genes encoding the IIC and IID proteins, respectively, were sequenced from genomic DNA and 11 mutants have either a mutation in the *ptnC* or in the *ptnD* gene (Table 1). Most mutations occurred within the *ptnC* gene and some of those mutations are identical. IL1403 mutants 9 and 12 contain mutations after residue 38, whereas mutants 7 and 11 have mutations after residue 81 of the IIC protein. These mutations resulted in severely truncated IIC proteins of 66 and 98 amino acids, respectively. Other significant mutations in the *ptnC* gene were detected in mutant 4, which has a 541-bp deletion of the 5′ part of the *ptnC* gene, and mutant 6, which has residues 41–53 deleted in IIC. The man-PTS mutations of mutants 4, 6, 7, 9, 11, and 12 will most likely alter the overall structure of the transporter. A much smaller deletion in the IIC protein was seen in mutant 8 with three deleted residues (residues 45–47), whereas mutant 3 contains a missense mutation (D25Y). Only three IL1403 mutants were found with mutations for the *ptnD* gene. A mutation after residue 206 of the IID protein in mutant 1 results in a truncated protein of 223 amino acids, whereas mutant 2 contains a missense mutation (N28K) and mutant 10 has residue 145 deleted. No mutations in the *ptnC* or *ptnD* genes were detected in mutant 5.

**TABLE 1:** Mutations in bacteriocin-resistant *L. lactis* IL1403 mutant strains

Mutant strain	Nucleotide mutation	Amino acid change
Mutant 1	*ptnD*: A620 deletion	Premature termination of PtnD after residue 206
Mutant 2	*ptnD*: C84A	N28K in PtnD
Mutant 3	*ptnC*: G73T	D25Y in PtnC
Mutant 4	541-bp deletion of 5′ *ptnC*	PtnC deleted
Mutant 5	No mutation in *ptnC* and *ptnD*	No mutation in PtnC and PtnD
Mutant 6	*ptnC*: codons 41–53 deleted	Residues 41–53 deleted in PtnC
Mutant 7	*ptnC*: C245-T290 deletion	Premature termination of PtnC after residue 81
Mutant 8	*ptnC*: codons 45–47 deleted	Residues 45–47 deleted in PtnC
Mutant 9	*ptnC*: A115-C181 deletion	Premature termination of PtnC after residue 38
Mutant 10	*ptnD*: codon 145 deleted	F145 deleted in PtnD
Mutant 11	*ptnC*: C245-T290 deletion	Premature termination of PtnC after residue 81
Mutant 12	*ptnC*: A115-C181 deletion	Premature termination of PtnC after residue 38

**Fig 1:**
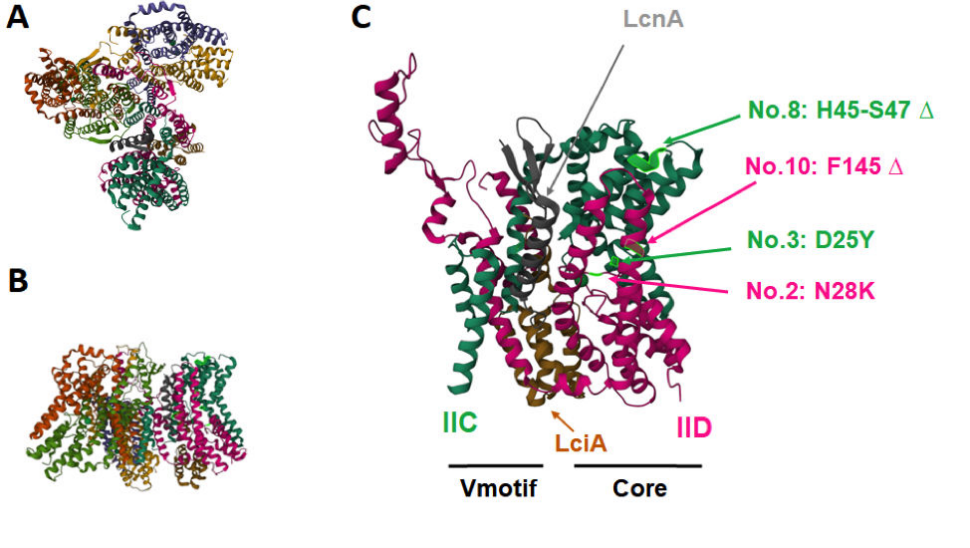
Structure of IIC and IID of the man-PTS of *L. lactis* IL1403 in complex with lactococcin A (LcnA) and lactococcin A immunity protein (LciA) (PDB: 8HFS). (**A**) Man-PTS trimer viewed from the top; (**B**) man-PTS trimer viewed from the side and parallel to the membrane; and (**C**) IICIID protomer showing the position of mutations in mutant no. 2, 3, 8, and 10. Green ball depicts sugar.

Recently, the structure of man-PTS of *L. lactis* IL1403 in complex with lactococcin A and the immunity protein for lactococcin A was elucidated (25). This enabled us to locate the various minor mutations we observed in our IL1403 mutants that are resistant to lactococcin A (Fig. 1). None of the minor man-PTS mutations are located in the vicinity where lactococcin A-maltose-binding fusion protein is inserted in the cleft between the Vmotif and Core domain of man-PTS according to the reported cryo-EM structure. The mutations of mutants 2, 3, 8, and 10 are all located in the Core or sugar transport domain of man-PTS. Interestingly, the missense mutations of mutants 2 and 3 are very near the pocket inside the Core domain where the sugar is likely to be bound (Fig. 1). This means that mutations in the sugar-binding pocket may somehow block the antibacterial activity of lactococcin A. The reported cryo-EM structure of the lactococcin A fusion protein bound to the receptor (25) does not visibly account for these observations with the parent bacteriocin.

In this study, only the man-PTS genes have been sequenced. Therefore, it cannot be ruled out, as is likely the case for mutant 5, that other mutations outside the man-PTS gene cluster have been generated, which contributed to the observed resistance of IL1403 mutants to lactococcin A.

### Resistance levels of *L. lactis* IL1403 mutants to lactococcin A and garvicin Q

**Fig 2:**
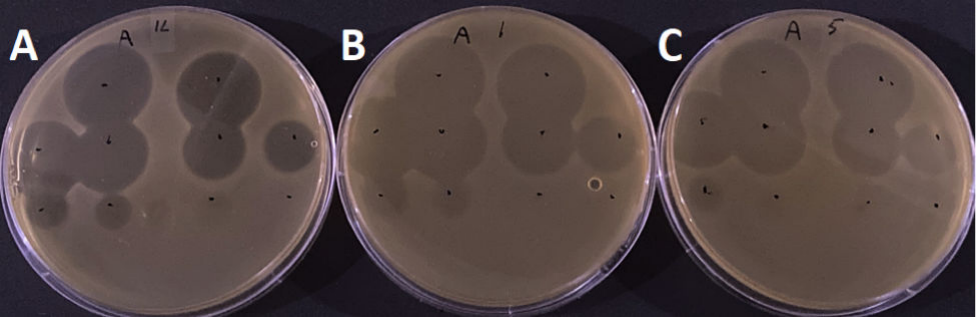
Spot-on-lawn assays showing antibacterial activity of lactococcin A against WT *L. lactis* IL1403 (**A**), IL1403 mutant 1 (**B**), and IL1403 mutant 5 (**C**) in APT medium. Lactococcin A was serial diluted twofold from top to bottom and left to right starting at 400 µM.

The 12 *L*. *lactis* IL1403 mutants were grown in All Purpose Tween (APT) medium and tested for their level of resistance to lactococcin A using spot-on-lawn assays (Table 2). Wild-type (WT) *L. lactis* IL1403 was used as a control strain. Lactococcin A was able to inhibit WT IL1403 at 3.2 µM. However, even at 400 µM, no inhibition zones were detected for any of the mutants, except for mutants 1, 3, and 5. Mutant 3 only showed growth inhibition at the highest level of lactococcin A used (400 µM), whereas opaque inhibition zones by up to 3.2 µM were observed for mutants 1 and 5 (Fig. 2). Apparently, IL1403 mutants 1 and 5 show some sensitivity to lactococcin A but are still highly resistant.

**TABLE 2:** Minimum inhibitory concentrations (μM) of bacteriocins against *L. lactis* IL1403 wild-type and mutant strains grown in APT and GM17 media

Strain	Lactococcin A (APT)	Garvicin Q (APT)	Garvicin Q (GM17)
Wild type	3.2	3.2	0.8
Mutant 1	3.2^*a*^	3.2^*a*^	0.8^*a*^
Mutant 2	>400	>400	400
Mutant 3	400	>400	200
Mutant 4	>400	>400	>400
Mutant 5	3.2^*a*^	3.2^*a*^	0.8^*a*^
Mutant 6	>400	>400	>400
Mutant 7	>400	>400	>400
Mutant 8	>400	>400	>400
Mutant 9	>400	>400	>400
Mutant 10	>400	>400	200
Mutant 11	>400	>400	>400
Mutant 12	>400	>400	>400

^*a*^ Inhibition zones are opaque.

To test whether the 12 *L*. *lactis* IL1403 mutants are cross-resistant to other bacteriocins, we expressed and isolated garvicin Q using the simplified “sandwiched” SUMO-peptide-intein fusion protein strategy also used for the isolation of lactococcin A (29). This method makes use of an expression system that produces a fusion protein with His-tags to the SUMO, as well as to the intein. The expression of a “sandwiched” bacteriocin not only prevents the degradation of the peptide but the His-tags at both ends of the fusion protein also simplify its isolation. Mature garvicin Q shares no significant sequence homology with mature lactococcin A and has a much wider inhibition spectrum (15, 30). The SUMO-garvicin Q-intein fusion protein was isolated and purified, and after removal of the SUMO and intein tags, it yielded ~1 mg of garvicin Q per liter of *E. coli* culture used. Matrix-assisted laser desorption ionization-time of flight mass spectrometry (MALDI-TOF MS) confirmed the presence and purity of the peptide. An average molecular mass of 5,344.0 Da was observed for the peptide (data not shown). When WT *L. lactis* IL1403 and the 12 IL1403 mutants were tested in APT medium for sensitivity to garvicin Q using spot-on-lawn assays, the results were similar to those obtained for lactococcin A (Table 2). Garvicin Q inhibited the growth of WT *L. lactis* IL1403, as well as those of mutants 1 and 5 at 3.2 µM. However, the inhibition zones for mutants 1 and 5 were again opaque indicating some sensitivity to garvicin Q (data not shown). All other mutants did not show any inhibition zones by garvicin Q at 400 µM. This means that only mutant 3 showed a slightly different inhibition result between the two bacteriocins (Table 2). Spot-on-lawn assays using garvicin Q were also performed with WT IL1403 and IL1403 mutants grown in an M17 medium supplemented with glucose (GM17) instead of APT. Interestingly, a fourfold increase in sensitivity to garvicin Q was observed for IL1403 (Table 2) showing that the growth medium can play a role in sensitivity to bacteriocins. Garvicin Q inhibited WT IL1403 and mutants 1 and 5 at 0.8 µM, although the inhibition zones for the two mutants were again opaque (data not shown). Mutants 3 and 10, and mutant 2, now showed inhibition by garvicin Q at 200 and 400 µM, respectively.

Spontaneous *L. lactis* IL1403 mutants that became resistant to garvicin Q have been described before (15). Most of those mutants contained frameshift mutations in the *ptnC* and *ptnD* genes. One mutant had a mutation identical to our mutant 10 (deletion of F145 in IID). Another mutant had residues 12–15 (IVVA) in IIC replaced by three different residues (LDT) that are located in the Core domain (15). Mutants were also isolated that contained a missense mutation in IID (P123H), which is located near the cleft between the Vmotif and Core domain, where the bacteriocin is proposed to be inserted (15).

**Fig 3:**
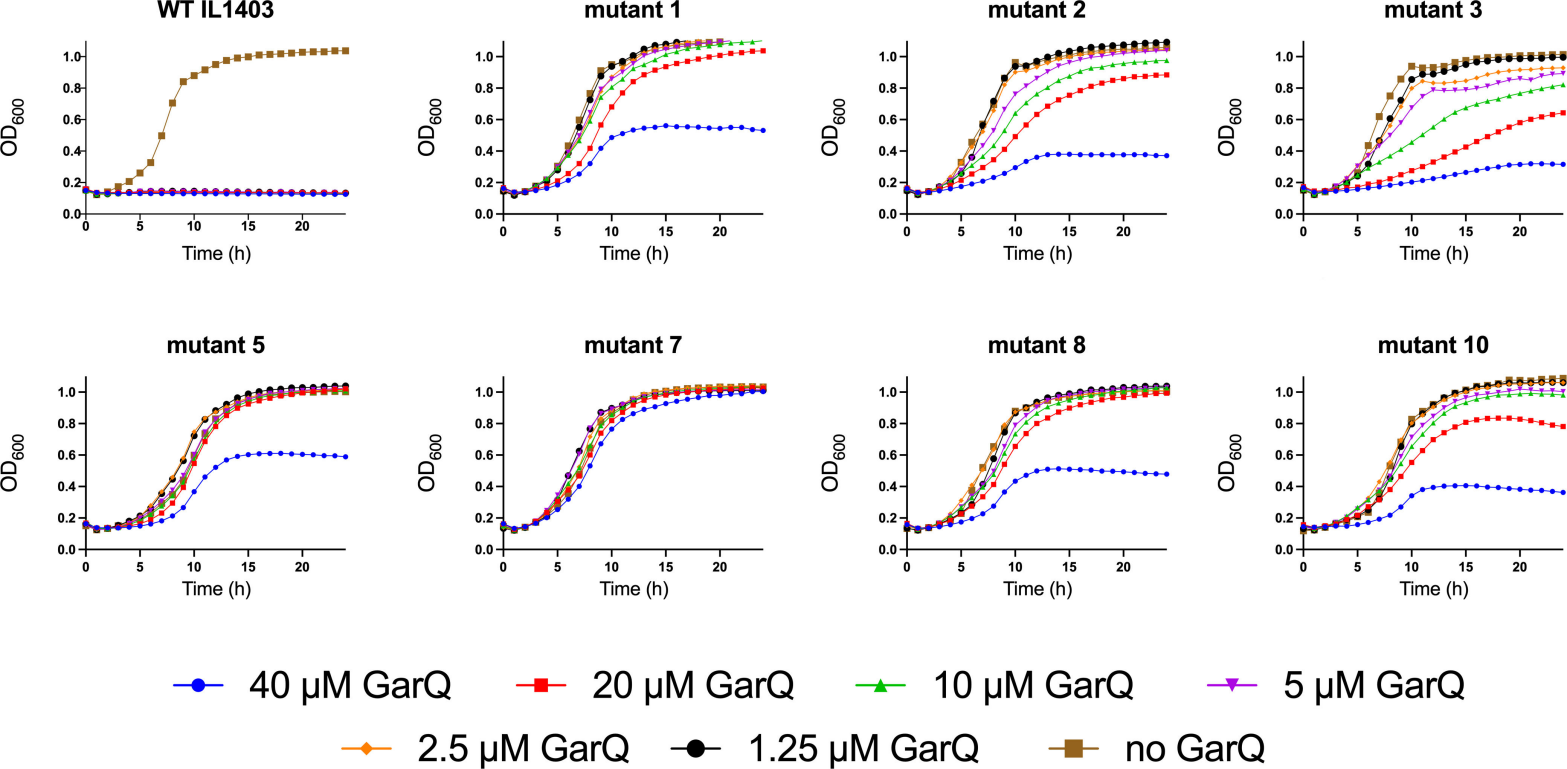
Growth curves of WT *L. lactis* IL1403 and IL1403 mutants 1, 2, 3, 5, 7, 8, and 10 in the presence of various concentrations of garvicin Q (GarQ) in APT medium.

In order to investigate whether there is a difference in bacteriocin sensitivity when cells are grown in liquid media compared to solid agar, growth curve experiments were performed using WT IL1403 and various IL1403 mutants in the presence of garvicin Q in liquid APT (Fig. 3). Whereas WT IL1403 showed no growth at 1.25 µM garvicin Q, mutant 7 was barely inhibited in its growth by 40 µM garvicin Q. The growth of mutants 2, 3, and 10 was affected by 20 µM garvicin Q and, in the case of mutants 2 and 3, even by 10 µM garvicin Q. These results show that mutants with relatively small mutations, such as missense mutations in mutants 2 and 3, or a codon deletion in mutant 10, still have some sensitivity to the bacteriocin. Mutants 1 and 5 are only significantly affected by garvicin Q in liquid media at 40 µM, confirming that these mutants are fairly resistant to the bacteriocins tested and explaining the opaque nature of the inhibition zones in spot-on-lawn assays.

### Sugar metabolism of *L. lactis* IL1403 mutants

**Fig 4:**
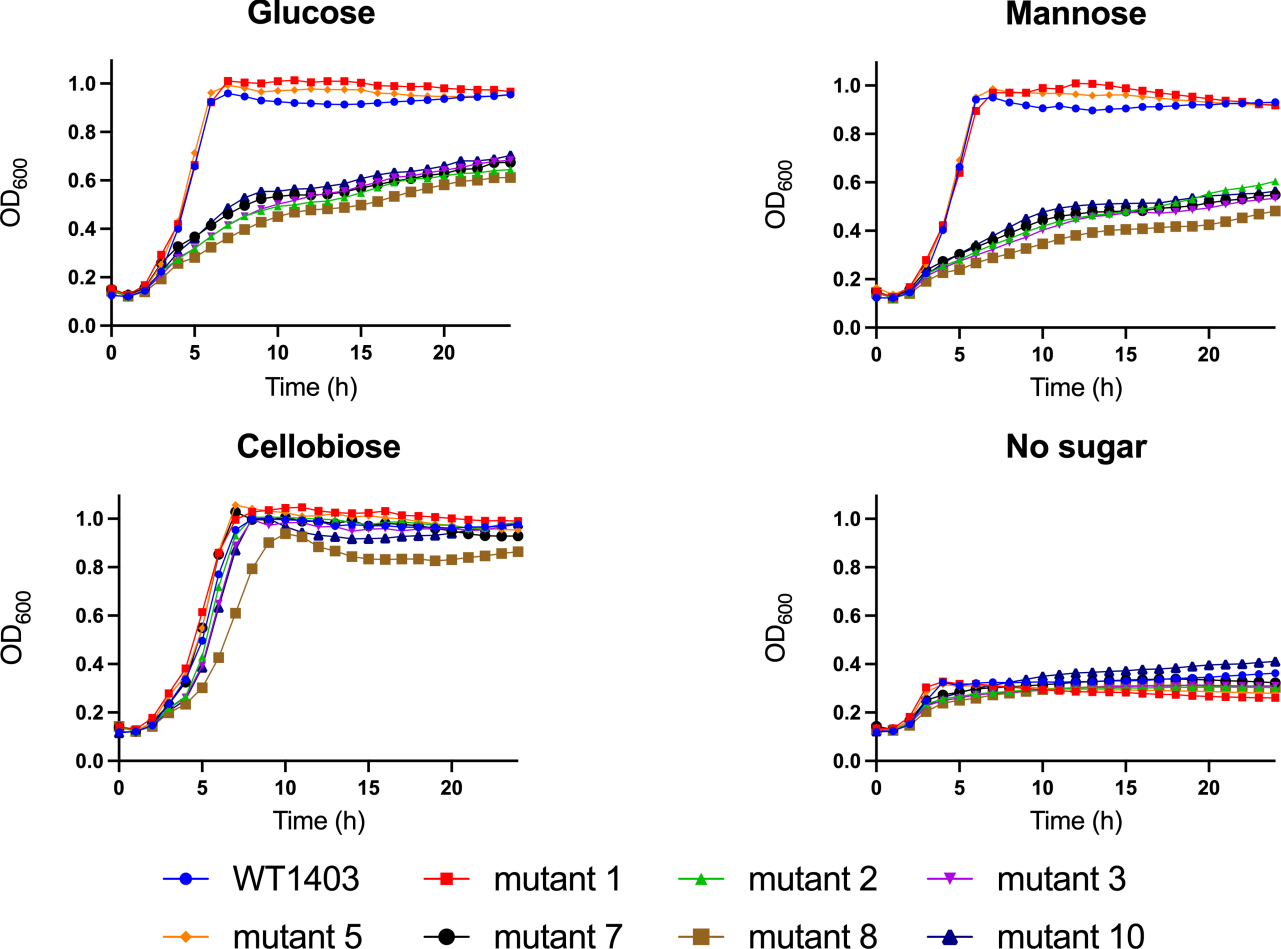
Growth curves of WT *L. lactis* IL1403 and IL1403 mutants 1, 2, 3, 5, 7, 8, and 10 in M17 medium supplemented with glucose, mannose, or cellobiose, and M17 medium without sugar as control.

To investigate whether the various mutations of the man-PTS affect the growth rate of IL1403 on sugars that utilize the man-PTS, the growth of IL1403 mutants was monitored in an M17 medium supplemented with either glucose or mannose and compared with growth in M17 with a non-man-PTS sugar such as cellobiose or in M17 without a carbon source. In *L. lactis* IL1403, cellobiose is not taken up by man-PTS but by a different cellobiose-specific PTS and thus was used here as a positive control (31). Figure 4 shows that the growth rate of all mutants, except for mutants 1 and 5, was negatively affected when cells were grown in M17 supplemented with mannose compared to the WT IL1403 strain. A similar result was obtained when various mutants were grown in M17 supplemented with glucose. Again, only mutants 1 and 5 had similar growth curves as WT IL1403, whereas other mutants had a lower growth rate as the WT strain. It is remarkable that mutant 1 seems to have a functional man-PTS despite the deletion of the C-terminal end of IID. Given the fact that mutant 5 has an intact IICIID permease and is able to transport mannose and glucose implies that the acquired bacteriocin resistance in this mutant is not due to the downregulation of the *ptnCD* genes. As expected, little difference in growth rate was seen between WT and mutants of IL1403 when cells were grown in M17 supplemented with cellobiose, and growth was impaired for all strains when grown in M17 without a carbon source (Fig. 4). These results reveal that the missense mutations in the Core domain of the man-PTS of mutants 2 and 3 not only gave rise to bacteriocin resistance but also impeded the uptake of sugars. The observation that *L. lactis* IL1403 mutants still show some growth in the presence of glucose despite having a man-PTS that is no longer able to take up sugars can be explained by the presence of other sugar transporters. Glucose can utilize the non-PTS low-affinity permease GlcU in *L. cremoris* NZ9000 and it is postulated that this permease is also present in *L. lactis* IL1403 (32).

### Antibacterial activity of MBP-bacteriocin fusion proteins

**Fig 5:**
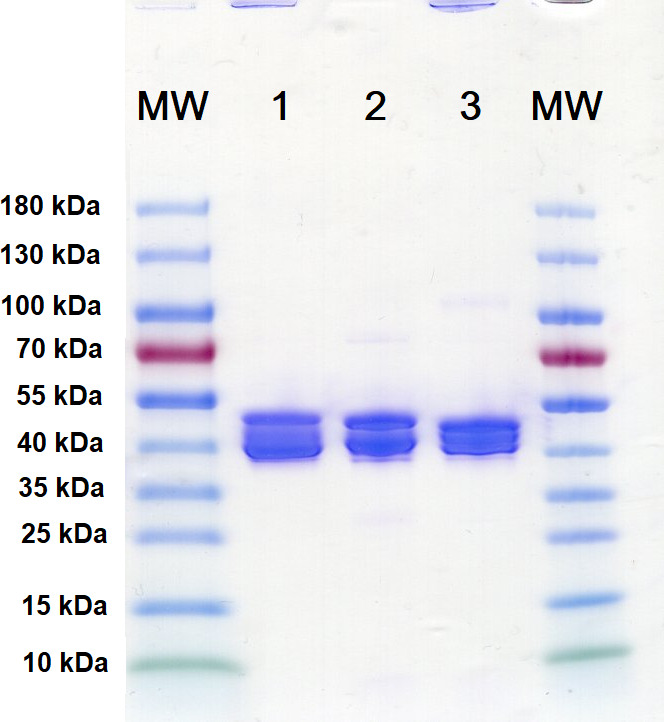
SDS-PAGE of MBP-bacteriocin fusion proteins. MW, molecular weight marker; 1, MBP-lactococcin A; 2, MBP-garvicin Q; and 3, MBP-pediocin PA-1.

**Fig 6:**
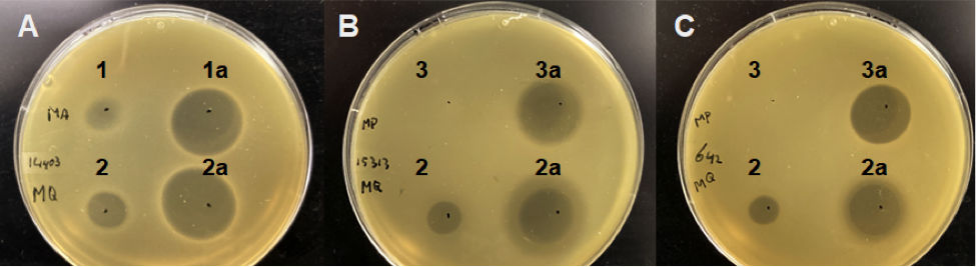
Spot-on-lawn assays showing the antibacterial activity of MBP-bacteriocin fusion proteins against *L. lactis* IL1403 (**A**), *L. monocytogenes* ATCC 15313 (**B**), and *L. monocytogenes* HPB 642 (**C**) in APT medium. 1, MBP-lactococcin A; 1a, MBP-lactococcin A digested with protease Xa; 2, MBP-garvicin Q; 2a, MBP-garvicin Q digested with protease Xa; 3, MBP-pediocin PA-1; and 3a, MBP-pediocin PA-1 digested with protease Xa.

With the exception of leaderless bacteriocins (33), class II bacteriocins are produced as precursors containing double-glycine-type leader peptides or signal peptides at the N-terminus of the bacteriocin that access a dedicated transport pathway or the general secretion pathway of the bacterial cell, respectively (34). The presence of a leader sequence reduces the antibacterial activity of these precursors and might play a role in inactivating the bacteriocin (35). The precursor for carnobacteriocin B2 was shown to be ~125 times less active than the mature carnobacteriocin B2 without the 18-amino acid leader peptide (36). Nevertheless, some antibacterial activity can be detected when bacteriocin precursors are overexpressed in *E. coli* (37 – 39). The cryo-EM structures of man-PTS in combination with bacteriocins were all obtained using bacteriocins fused at the N-terminus with a MBP (23 – 25). Although some antibacterial activity was reported for these fusion proteins, it raises the question of whether antibacterial activity is reduced when bacteriocins are fused to MBP. Therefore, three MBP-bacteriocin fusion proteins, using lactococcin A, garvicin Q, and pediocin PA-1, were constructed, overexpressed, and isolated from *E. coli*. As can be seen in Fig. 5, some degradation of the MBP-bacteriocin fusion proteins occurred and shows that truncation of the bacteriocins during expression in *E. coli* is not specific to the affinity tag that is being used (26). Spot-on-lawn assays in APT revealed that the minimum concentration of MBP-lactococcin A and MBP-garvicin Q to inhibit *L. lactis* IL1403 is 40 µM, which is higher than observed for the mature bacteriocins. Indeed, when these MBP-bacteriocin fusion proteins were digested with Factor Xa protease to release the bacteriocins from the fusion proteins, much larger zones of inhibition were observed (Fig. 6A). *L. monocytogenes* strains ATCC 15313 and HPB 642 were both inhibited by MBP-garvicin Q at 40 µM, but not by MBP-pediocin PA-1 (Fig. 6B and C). No inhibition of *L. monocytogenes* was observed when MBP-pediocin PA-1 was spotted twice on the same spot to mimic a bacteriocin concentration of 80 µM (data not shown). On the other hand, large zones of inhibition of *L. monocytogenes* were achieved after the digestion of MBP-pediocin PA-1 with Factor Xa protease, indicating the sensitivity of these strains to mature pediocin PA-1 (Fig. 6B and C). Moreover, an MBP-pediocin PA-1 solution of 40 µM that was digested by the protease could be diluted up to 16-fold and still inhibit both *Listeria* strains (data not shown). This suggests that the MBP-pediocin PA-1 fusion protein is more than 32-fold less active than mature pediocin PA-1. It is unclear why our inhibitory results with the MBP-pediocin PA-1 seem to differ from those reported previously (23). However, it raises the issue of whether derivatives of compounds that are used for modeling studies need to reflect the natural compounds with respect to their biological activity.

### Conclusion

Almost all of the spontaneous lactococcin A-resistant IL1403 mutants that were isolated from the APT medium in this study contain mutations in the *ptnC* or *ptnD* genes encoding the IIC and IID proteins of the man-PTS. Most of the mutants contain a greatly disrupted man-PTS and those mutants are highly resistant to lactococcin A and garvicin Q. Interestingly, none of the more minor mutations, such as those shown for mutants 2, 3, 8, and 10, were located where the bacteriocin is proposed to be inserted into the man-PTS based on cryo-EM studies with bacteriocin fusion proteins. Instead, these mutations were all found in the Core domain of man-PTS, which is responsible for sugar transport. These mutants lost the ability to transport mannose or glucose but still showed some sensitivity to high concentrations of bacteriocin, suggesting that man-PTS is still present in these cells. These results seem to indicate that a minor change in the sugar-binding pocket changes the conformation of the man-PTS to such a degree that it manages to block the pore formation of the bacteriocin. It is as yet unclear how this conformational change affects the interaction between the bacteriocin and man-PTS. However, it illustrates that the mechanism of pore formation by bacteriocins that target the man-PTS still is not fully elucidated.

## MATERIALS AND METHODS

### Bacterial strains and media

*L. lactis* IL1403 was grown in All Purpose Tween medium (Difco) or M17 (Difco) medium supplemented with either glucose (GM17), mannose (MM17), or cellobiose (CM17) (0.5%, wt/vol) at 30°C. *L. monocytogenes* strains ATCC 15313 and HPB 642 were grown in APT medium at 30°C. NEB 10-beta *E. coli* (New England Biolabs) and *E. coli* BL21(DE3) (New England Biolabs) were grown in Luria Broth (LB; Difco) at 37°C containing 100 µg/mL ampicillin unless specified differently. Soft agar and agar plates were prepared by the addition of agar (Difco) at 0.7% (wt/vol) and 1.5% (wt/vol), respectively, to the medium.

### Isolation of lactococcin A-resistant mutants of *L. lactis* IL1403

*L. lactis* IL1403 mutants resistant to lactococcin A were obtained by isolating tiny bacterial colonies that appeared over time in inhibition zones produced by lactococcin A on APT agar plates overlaid with soft APT agar inoculated with IL1403. The mutant IL1403 colonies from the inhibition zones were streaked on fresh APT agar plates and incubated overnight. Single colonies that appeared the next day were used to inoculate 5 mL of APT medium. Fully grown cultures were subsequently stored at −70°C.

### Genomic DNA isolation and sequencing of man-PTS genes

Genomic DNA from *L. lactis* IL1403 mutants was isolated using the DNeasy Blood & Tissue Kit from Qiagen and used as a template to amplify the *ptnC* and *ptnD* genes. DNA was amplified using forward primer MVB313 (5′-ATGCTCGTCTGATATTCTGATT-3′) and reverse primer MVB316 (5′-TTATACCAAATCGATTACATGAT-3′). In case no PCR product was detected, forward primer MVB322 (5′-CGCTCTTGCAACAATCGAAG-3′) and reverse primer MVB323 (5′-GTCACCGATACCGGCAAGA-3′) were used for the amplification of *ptnC* and *ptnD*. Primer sequences were based on the genomic DNA sequence of *L. lactis* IL1403 (GenBank: CP033607.1). The resulting PCR products were purified with the GeneJet PCR Purification kit (ThermoScientific) and sequenced at the nucleotide level.

### Lactococcin A and garvicin Q expression and isolation

The gene cloning, expression, isolation, and purification of lactococcin A have been described previously (29). A similar procedure was followed to overexpress, isolate, and purify garvicin Q. Changes to the protocol included the use of primers specific to the garvicin Q structural gene (*garQ*) and having sequence tails for ligation with PacI-digested pSPIH6 vector (Addgene; plasmid #190-676). The primers used were the forward primer GarQ_SUMO_for (5′-GCTCACAGAGAACAGATTGGTGGTGAATATCACCTAATGAACGG-3′) and reverse primer GarQ_intein_rev (5′-AGTGCATCTCCCGTGATGCAGTGCTGCGGACCAAAACCTG-3′), and they were designed to amplify the *garQ* gene without the leader peptide coding sequence. *E. coli* BL21(DE3) cells carrying the appropriate construct for garvicin Q expression were cultured overnight with shaking (225 rpm; 37°C) in LB and then inoculated to a final concentration of 4% in 1 L of Terrific Broth medium (Invitrogen) supplemented with 4% glycerol (wt/vol) and ampicillin. Cells were cultured to an optical density (OD_600_) of 0.7 at 37°C, and then garvicin Q expression was induced by adding isopropyl β-d-1-thiogalactopyranoside (IPTG) at a final concentration of 0.5 mM and cells were grown for ca. 28 h (225 rpm; 15°C). Further isolation of overproduced garvicin Q followed the procedure described by Lamer and Vederas (29), except that elution of the fusion protein from the Ni-NTA resin (Qiagen) was done with elution buffer (50 mM NaH_2_PO_4_ and 300 mM NaCl, pH 8.5) containing imidazole only at a concentration of 500 mM. The resulting peptide was further purified using semi-preparative high performance liquid chromatography (HPLC) (Agilent) with 0.1% trifluoroacetic acid (TFA) and 0.1% TFA in acetonitrile (ACN) as eluents. The ACN solvent was first set at 20% for 6 min, gradually increased to 55% for 9 min, and raised to 100% for 4 min. The peptide was eluted at 47% ACN concentration at 12 min. The column used for HPLC was a ZORBAX 300 C18, 9.4 × 250 mm, 5 µm (Agilent). Purity and fraction masses were controlled by MALDI-TOF MS on a Perspective Biosystems Voyager Elite spectrometer (AB Sciex). The fraction containing the purified peptide with a calculated molecular weight of 5,344.0 Da was lyophilized and resuspended in an appropriate volume of aqueous 0.1% TFA to obtain a final concentration of 400 µM.

### MBP-bacteriocin construction, expression, and isolation

The DNA sequence encoding mature pediocin PA-1 (uniprot ID P29430) was codon optimized for *E. coli* (Integrated DNA Technologies), synthesized, and cloned into the vector pMAL-c5X using XmnI and HindIII restriction sites, respectively (GenScript), to construct the maltose-binding protein fusion gene for pediocin PA-1 (*MBP-pedA*). The plasmid was then transformed into *E. coli* BL21(DE3) cells.

The gene for *E. coli* codon-optimized, mature lactococcin A was synthesized and cloned into a pET-SUMO vector previously (26) and was PCR amplified here with primers MBP-LcnA-F (5′-TCGGGATCGAGGGAAGGAAACTGACCTTTATTCA-3′) and MBP-LcnA-R (5′- ATCCGAATTCTGAAATTTAATGATGCAGGCCAAAG-3′) and then inserted into an XmnI-digested pMAL-c2X vector using NEBuilder HiFi DNA Assembly (New England Biolabs). The resulting vector containing the maltose-binding protein fusion gene for lactococcin A (*MBP-lcnA*) was then transformed into NEB 10-beta *E. coli* cells and grown on LB plates. Plasmid DNA from selected colonies was purified (GeneJET Plasmid Miniprep Kit; ThermoScientific) and sequenced using sequencing primers pMAL-F (5′-TCAACGCCGCCAGCGGTC-3′) and pMAL-R (5′- TTTCCCAGTCACGACGTTGT-3′) to confirm the nucleotide sequence. The plasmid was then transformed into *E. coli* BL21(DE3) cells.

The gene for *E. coli* codon-optimized, mature garvicin Q was synthesized and cloned into the pSPIH6 vector as described above. The *garQ* gene was PCR amplified with primers MBP-GarQ-F (5′-TCGGGATCGAGGGAAGGGAATATCACCTAATGAAC-3′) and MBP-GarQ-R (5′-ATCCGAATTCTGAAATTTAGTGCTGCGGACCAAAA-3′) and then inserted into pMAL-c2X and transformed into *E. coli* BL21(DE3) as described above to create the maltose-binding protein fusion gene for garvicin Q (*MBP-garQ*).

For the overexpression of MBP-bacteriocin fusion proteins, *E. coli* BL21(DE3) cultures containing the appropriate plasmid encoding the MBP-bacteriocin fusion gene were inoculated into 50 mL of LB media. The cells were grown by shaking at 225 rpm overnight at 37°C. The next day, 20 mL of the overnight culture was added to 500 mL of LB media, and cells were grown to an OD_600_ of 0.7 at 37°C. Protein expression was then induced by the addition of IPTG to a final concentration of 0.5 mM, and the flasks were then shaken at 225 rpm for 4 h at 37°C. The cells were harvested by centrifugation (5,000 × *g*, 10 min, 4°C), and the pellets were stored at −70°C.

Frozen pellets of the cells expressing MBP-bacteriocin fusion proteins were resuspended in ice-cold Tris lysis buffer (20 mM Tris and 200 mM NaCl, pH 7.5). Cells were lysed by sonication while kept on ice. DNase I (Thermo Scientific, 1 U) was added, and the lysate was kept on ice for 15 min with occasional mixing. The cellular debris was removed by centrifugation (20,000 × *g*, 30 min, 4°C), and the soluble supernatant was loaded onto a pre-washed amylose resin (New England BioLabs) column (~12 mL of resin/1 L culture media) at 4°C. The flow through was passed over the column three times. The column was then washed with ~100 mL of Tris lysis buffer with 1 mM EDTA and 1 mM dithiothreitol was added. The column was eluted in ~2 mL fractions by the addition of elution buffer (20 mM Tris, 200 mM NaCl, and 10 mM maltose). Fractions were analyzed for the presence of protein by measuring the absorbance at 280 nm using a Nanodrop spectrophotometer. Protein molecular weights and extinction coefficients were predicted using ExPASY. Protein-containing fractions were pooled, and then the buffer was exchanged for Tris lysis buffer, and the sample was concentrated to ~50 µM using an Amicon Ultra Centrifugal Filter (10 kDa MWCO, 15 mL). The final concentration of the purified MBP-bacteriocin fusion proteins was confirmed using absorbance at 280 nm, as well as with a Bradford assay, and the purity was confirmed using sodium dodecyl-sulfate polyacrylamide gel electrophoresis analysis.

### Bacteriocin assays

To determine the sensitivity of bacterial strains to bacteriocins, either spot-on-lawn assays or cell growth inhibition assays using microtiter plates were used.

For spot-on-lawn assays, bacterial cultures were grown overnight in GM17 or APT. Soft GM17 or APT agar (5 mL) was inoculated with overnight cultures (2%, vol/vol) and overlaid on solid GM17 or APT agar plates, respectively. Stock solutions of 400 µM were made of lactococcin A and garvicin Q, and 40 µM for the fusion proteins MBP-lactococcin A, MBP-garvicin Q, and MBP-pediocin PA-1. A twofold serial dilution of the bacteriocins and fusion proteins was made, and 10 µL of each concentration of bacteriocin or fusion protein was spotted onto the agar plate. The inhibitory activity of the fusion proteins was also tested after a 2 h digestion at room temperature of the fusion proteins with Factor Xa Protease (New England Biolabs) (0.05 mg/mL). After overnight incubation, the agar plates were examined for zones of inhibition.

The sensitivity of *L. lactis* IL1403 mutants to garvicin Q at concentrations in the range of 40–0.04 µM in liquid cultures was determined in a BioTek Epoch Microplate Spectrophotometer (Agilent, USA) with serial twofold dilutions of bacteriocin in APT medium. The data in the graphs are presented as means of at least three replicates, and the errors (SD) did not exceed 20%.

### Growth tests

Growth assays of WT *L. lactis* IL1403 and its lactococcin A-resistant mutants were performed in CM17, GM17, MM17, or M17 medium with no sugar added using a BioTek Epoch Microplate Spectrophotometer. OD_600_ was monitored during a 24-h incubation at 30°C at 0.5 h intervals. The data in the graphs are presented as means of at least three replicates, and the errors (SD) did not exceed 20%.
